# Targeting transitioning lung monocytes/macrophages as treatment strategies in lung disease related to environmental exposures

**DOI:** 10.1186/s12931-024-02804-3

**Published:** 2024-04-09

**Authors:** Aaron D. Schwab, Todd A. Wyatt, Grace Moravec, Geoffrey M. Thiele, Amy J. Nelson, Angela Gleason, Oliver Schanze, Michael J. Duryee, Debra J. Romberger, Ted R. Mikuls, Jill A. Poole

**Affiliations:** 1 Division of Allergy & Immunology, University of Nebraska Medical Center, Omaha, NE USA; 2 Veterans Affairs Nebraska-Western Iowa Health Care System, Research Service, Omaha, NE USA; 3 Division of Pulmonary, Critical Care & Sleep, University of Nebraska Medical Center, Omaha, NE USA; 4 Department of Environmental, Agricultural and Occupational Health, College of Public Health, University of Nebraska Medical Center, Omaha, NE USA; 5 Division of Rheumatology and Immunology, Department of Internal Medicine, College of Medicine, University of Nebraska Medical Center, Omaha, NE USA

**Keywords:** Endotoxin, Organic dust, Immunology, Inflammation, Lung disease, Monocytes

## Abstract

**Background:**

Environmental/occupational exposures cause significant lung diseases. Agricultural organic dust extracts (ODE) and bacterial component lipopolysaccharide (LPS) induce recruited, transitioning murine lung monocytes/macrophages, yet their cellular role remains unclear.

**Methods:**

CCR2 RFP^+^ mice were intratracheally instilled with high concentration ODE (25%), LPS (10 μg), or gram-positive peptidoglycan (PGN, 100 μg) for monocyte/macrophage cell-trafficking studies. CCR2 knockout (KO) mice and administration of intravenous clodronate liposomes strategies were employed to reduce circulating monocytes available for lung recruitment following LPS exposure. Lung tissues and bronchoalveolar lavage fluid (BALF) were collected. Pro-inflammatory and/or pro-fibrotic cytokines, chemokines, and lung extracellular matrix mediators were quantitated by ELISA. Infiltrating lung cells including monocyte/macrophage subpopulations, neutrophils, and lymphocytes were characterized by flow cytometry. Lung histopathology, collagen content, vimentin, and post-translational protein citrullination and malondialdehyde acetaldehyde (MAA) modification were quantitated. Parametric statistical tests (one-way ANOVA, Tukey’smultiple comparison) and nonparametric statistical (Kruskal–Wallis, Dunn’s multiple comparison) tests were used following Shapiro–Wilk testing for normality.

**Results:**

Intratracheal instillation of ODE, LPS, or PGN robustly induced the recruitment of inflammatory CCR2^+^ CD11c^int^CD11b^hi^ monocytes/macrophages and both CCR2^+^ and CCR2^−^ CD11c^−^CD11b^hi^ monocytes at 48 h. There were also increases in CCR2^+^ CD4^+^ and CD8^+^ T cells and NK cells. Despite reductions in LPS-induced lung infiltrating CD11c^int^CD11b^hi^ cells (54% reduction), CCR2 knockout (KO) mice were not protected against LPS-induced inflammatory and pro-fibrotic consequences. Instead, compensatory increases in lung neutrophils and CCL2 and CCL7 release occurred. In contrast, the depletion of circulating monocytes through the administration of intravenous clodronate (vs. vehicle) liposomes 24 h prior to LPS exposure reduced LPS-induced infiltrating CD11c^int^CD11b^hi^ monocyte-macrophage subpopulation by 59% without compensatory changes in other cell populations. Clodronate liposome pre-treatment significantly reduced LPS-induced IL-6 (66% reduction), matrix metalloproteinases (MMP)-3 (36%), MMP-8 (57%), tissue inhibitor of metalloproteinases (61%), fibronectin (38%), collagen content (22%), and vimentin (40%). LPS-induced lung protein citrullination and MAA modification, post-translational modifications implicated in lung disease, were reduced (39% and 48%) with clodronate vs. vehicle liposome.

**Conclusion:**

Highly concentrated environmental/occupational exposures induced the recruitment of CCR2^+^ and CCR2^−^ transitioning monocyte-macrophage and monocyte subpopulations and targeting peripheral monocytes may reduce the adverse lung consequences resulting from exposures to LPS-enriched inhalants.

**Supplementary Information:**

The online version contains supplementary material available at 10.1186/s12931-024-02804-3.

## Introduction

Environmental and occupational lung diseases remain a significant cause of pulmonary impairment worldwide [1]. Chronic respiratory diseases including chronic obstructive pulmonary disease (COPD), asthma, asthma-like syndrome, byssinosis, hypersensitivity pneumonitis, and pulmonary fibrosis have been associated with exposure to organic dusts. Organic dusts are comprised of particulate matter, components of bacteria, fungi, viruses, pollen, and fragments of animals and plants [2]. These exposures are common to agriculture and farming, the grain and food processing industry, waste and recycling facilities, the textile and cotton industry, woodworking, concentrated urban areas, flood- and water-damaged buildings, and more [3–7]. Retrospective analysis of the Global Burden of Disease database identified roughly 519,100 deaths and 13.6 million disability-adjusted life years in 2016 from chronic respiratory disease due to occupational airborne exposures [8]. The incidence and prevalence of interstitial lung disease increased from 1990 to 2019, with occupational exposure implicated as a leading risk factor despite increased awareness and implementation of preventative measures [9]. However, there remains a paucity of therapeutic options aimed at hastening recovery and/or preventing chronic disease resulting from these exposures.

Lipopolysaccharide (LPS) or endotoxin is found in the outer membrane of gram-negative bacteria [10–12] and acts as a well-defined component of many organic dusts and disease-causing environmental exposures. A role for gram-positive cell wall components such as peptidoglycans (PGN) in mediating lung disease following organic dust exposures has also been identified. Growing industrialization, intensified agricultural production, climate change, and the rising frequency and severity of extreme weather events have conspired to increase concentrations of aerosolized organic particulate matter and environmental endotoxins [13–16]. Organic dusts and its bacterial components engage innate immune signaling pathways (i.e., Toll-like receptors) to initiate airway inflammatory responses marked by the influx of neutrophils, lymphocytes, monocytes, and macrophages with a corresponding release of pro-inflammatory/fibrotic mediators (e.g., tumor necrosis factor (TNF)-α, interleukin (IL)-6, chemoattractants, extracellular matrix proteins). Organic dusts and LPS exposures also induce post-translational modifications in proteins, which may serve to increase inflammation and promote tissue fibrosis [17–20]. Although dust and bacterial component-induced lymphocytic lung aggregates were reduced in T and B cell depleted mice, many inflammatory consequences persisted. Thus, there is growing interest in the immunopathogenic role of distinct lung monocyte-macrophage subpopulations recruited and induced following environmental exposures [12, 21, 22]. CC motif chemokine receptor 2 (CCR2) is a critical facilitator of monocyte recruitment and activation via interaction with its high-affinity ligand CCL2 [23, 24]. CCR2^+^ monocytes play a critical role in the onset of inflammation and, upon reaching the inflamed tissue, differentiate into phenotypically and functionally distinct macrophages, capable of modulating inflammatory responses [25].

The objectives of this study were to first delineate the magnitude and distribution of CCR2^+^ (and CCR2^−^) monocytes and monocyte-derived macrophages trafficked to the lung following organic dust, LPS, and PGN exposures, then determine whether targeting these lung monocytes-macrophages would attenuate resulting pro-inflammatory and pro-fibrotic responses. We tested the hypotheses that pro-inflammatory and pro-fibrotic responses in lungs from mice exposed to inhaled LPS would be reduced in CCR2 deficient (knockout) mice compared to wild-type mice and that similar protection would be afforded in wild-type mice through the administration of intravenous clodronate liposome to deplete circulating monocytes. Understanding the role of recruited monocytes and monocyte-derived macrophages will provide fundamental knowledge of inflammatory processes following environmental exposures, but also potentially elucidate therapeutic targets to mitigate disease development in at-risk persons.

## Methods

### Environmental exposures

Lipopolysaccharide (LPS) from gram-negative *Escherichia coli* (O55:B5; Sigma, St. Louis, MO) served as the primary exposure in all experiments. The rationale was that LPS is commercially available and elicits a dose-dependent, reproducible pro-inflammatory lung response in humans and rodents. In studies of monocyte trafficking, comparisons were undertaken using peptidoglycan (PGN) from gram-positive *Staphylococcus aureus* (Sigma) and an aqueous solution of organic dust extract (ODE) prepared from swine confinement feeding facilities as previously described [26]. Briefly, settled surface dust (1 g) was incubated in sterile Hank’s Balanced Salt Solution (10 mL; Mediatech, Manassas, VA) for 1 h and centrifuged for 30 min at 2850 × g twice, with the final supernate filter-sterilized (0.22 um) to remove microorganisms and coarse particles. Stock ODE was batch prepared and stored at − 20 °C; aliquots were diluted for each experiment to a final concentration (vol/vol) of 25% in sterile phosphate buffered saline (PBS, pH 7.4; diluent). Endotoxin concentrations were determined using the limulus amebocyte lysate assay (Lonza, Walkersville, MD). Endotoxin levels averaged 1.308–2.616 μg (~ 10–50 EU) for 25% ODE. Prior mass spectrometry studies of ODE have revealed significant amounts of muramic acid (peptidoglycan marker) and 3-hydroxy fatty acids (endotoxin marker), but not ergosterol (fungi marker) as compared to house dust [26].

### Animal exposure model

C57BL/6 and homozygous CCR2^RFP/RFP^ (B6.129(Cg)-Ccr2^tm2.1lfc^/J) mice between 6 and 8 weeks of age were purchased from The Jackson Laboratory (Bar Harbor, ME). In this latter strain (#017586; RRID:IMSR_JAX:017586), a monomeric red fluorescent protein (RFP) sequence replaces the coding sequence of the *Ccr2* gene, abolishing gene function and thus referred to as CCR2 knockout (KO) mice. Mouse tail snips were collected and shipped for DNA extraction and targeted CCR2 genotyping (TransnetYX, Cordova, TN) to confirm CCR2 KO. To generate heterozygous CCR2^+/RFP^ mice in which CCR2 is functional yet marked by RFP expression, CCR2^RFP/RFP^ mice were bred to C57BL/6 wild-type (WT) mice. For experiments using heterozygous CCR2^+/RFP^ animals, male and female mice were utilized. For the CCR2 KO (and clodronate liposome studies), male mice were utilized, as male mice had increased inflammatory responses with less experimental variability following LPS exposure, consistent with prior studies [12, 27]. Mice were randomized, with AJN, AG, and animal facility staff aware of the randomization, whereas all other authors were blinded. To induce airway inflammation, mice were lightly sedated under isoflurane (VetOne, Boise, ID) and received one treatment with 50 μl of sterile saline (control), ODE (25%), LPS (10 μg), or PGN (100 μg) [28]. Animals were euthanized 48 h following exposure by isoflurane followed by exsanguination (right axillary blood collection). No respiratory distress, signs of stress, or significant weight loss (defined as > 20%) were observed throughout the exposure period.

### Clodronate-induced systemic monocyte/macrophage depletion

In separate studies, C57BL/6 WT mice were administered encapsulated clodronate liposomes intravenously to deplete systemic monocytes and recruited monocyte-derived macrophages [29–32]. Clodronate and control liposomes (Liposoma Technology, Amsterdam, Netherlands; 200 μl × 5 mg/ml) were injected into the tail vein one day prior to LPS and saline control exposure.

### Lavage fluid cells and lung homogenates

Bronchoalveolar lavage fluid (BALF) was collected using 3 × 1 mL PBS. Total cell numbers from the combined recovered lavage were enumerated using a BioRad TC 20 cell counter with differential cell counts determined from cytospin-prepared slides (cytopro cytocentrifuge, ELITech Group, Logan, UT) stained with Diff-Quick (Siemens, Newark, DE). Lung tissue homogenates were prepared by homogenizing lung samples (1/2 of right lungs) in 500 μl of sterile phosphate buffered saline (PBS) following removal of BALF and blood from the pulmonary vasculature. From cell-free lung tissue homogenates, levels of TNF-α, IL-6, murine neutrophil chemoattractant CXCL1, murine monocyte (and leukocyte) chemoattractants CCL2 and CCL7, and transforming growth factor (TGF)-β were quantitated by ELISA (R&D Systems, Minneapolis, MN) with minimal detectable difference (MDD) of 1.88, 1.6, 2.0, 0.3, 1.5, 31.3 pg/ml, respectively. Additionally, lung tissue homogenates were assessed for regulators of extracellular matrix deposition including matrix metalloproteinase (MMP)-3 and tissue inhibitor of metalloproteinase (TIMP)-1 (ELISA; R&D Systems; MDD of 0.125 and 0.031 ng/ml, respectively) as well as MMP-8 (ELISA; Abcam, Boston, MA; MDD of 0.053 ng/ml).

### Lung cell staining and flow cytometry

Following removal of BALF and blood from pulmonary vasculature, harvested lungs (1/2 of right lungs) were subjected to an automated dissociation procedure using a gentleMACS Dissociator instrument (Miltenyi Biotech, Auburn, CA). Viability of total lung cells was assessed by trypan blue exclusion and a LIVE/DEAD Fixable Blue Dead Cell Stain Kit (Invitrogen, Carlsbad, CA). Cell viability was > 99% with no differences by treatment group(s) (data not shown). Lung cells were incubated with CD16/32 (Fc Block, BioLegend, San Diego, CA) to minimize non-specific antibody staining, then stained with monoclonal antibodies against rat anti-mouse; CD45 (clone: 30-F11; BD Biosciences, Franklin Lake, NJ), CD11b (clone: M1/70; BD Biosciences and BioLegend), Ly6G (clone: 1A8; BD Biosciences), CD11c (clone: N418; Invitrogen), CD4 (clone: RM4-5; BD Biosciences), CD8 (clone: 53–6.7; BD Biosciences), CD19 (clone: 1D3; Invitrogen), hamster anti-mouse CD3e (clone: 145-2C11; BD Biosciences and BioLegend), mouse anti-mouse NK1.1 (clone: PK136; BD Biosciences or BioLegend), Ly6C (clone: HK1.4; BioLegend), and F4/80 (clone: QA17A29; BioLegend or clone: T45-2342; BD Biosciences). Cells were acquired on a BD LSRII Yellow/Green cytometer configured with 355-nm, 405-nm, 488-nm, 561-nm, and 633-nm lasers. Post-acquisition, data were exported and stored using the flow cytometry standard (FCS) 3.1 format and analyzed using FlowJo software version 10.8 (FlowJo, RRID:SCR_008520, Ashland, OR).

**Fig. 1:**
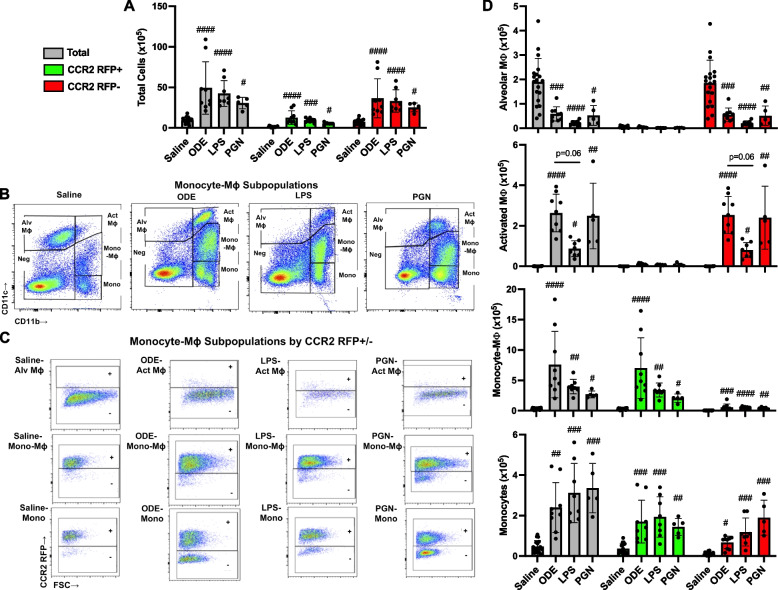
Inhalation of organic dust extract (ODE), lipopolysaccharide (LPS), and peptidoglycan (PGN) induce lung CCR2^+^ monocyte-macrophages (Mɸ) and monocytes. CCR2^RFP/+^ mice were exposed once to ODE (25%), LPS (10 μg), PGN (100 μg), or saline control and euthanized at 48 h. Scatter plots with bars depict mean with SD delineating cells as total (gray), CCR2^+^ (green), and CCR2^−^ (red). **A** Total lung cells enumerated. **B** Representative contour plot of the four monocyte (mono)-Mɸ subpopulations across groups based upon CD11c and CD11b expression after removal of neutrophils gated from live CD45^+^ cells after excluding debris and doublets. **C** RFP ± staining by exposure group and subpopulation. **D** CD11c^+^CD11b^lo^ alveolar (Alv) Mɸ, CD11c^+^CD11b^hi^ activated (Act) Mɸ, CD11c^int^CD11b^hi^ mono-Mɸ, and CD11c^−^CD11b.^hi^ monocytes determined by multiplying lung cell % population by total lung cells enumerated from lung sample. Statistical analyses were performed with Kruskal–Wallis with Dunn’s test for multiple comparisons (#*p* < 0.05, ##*p* < 0.01, ###*p* < 0.001, ####*p* < 0.0001) vs. respective saline. *N* = 19 (saline), 9 (ODE), 8 (LPS), 5 (PGN)

The gating strategies for Ly6G^+^ neutrophils, CD11c^+^CD11b^lo^ alveolar (Alv) macrophages (Mɸ), CD11c^+^CD11b^hi^ activated (Act) alveolar Mɸ, CD11c^int^CD11b^hi^ recruited/transitioning monocytes-Mɸ, CD11c^−^CD11b^hi^ monocytes, CD3^+^CD4^+^ T cells, CD3^+^CD8^+^ T cells, CD19^+^ B cells, and NK cells were performed as previously reported [12, 17, 18] with associated RFP^+^ gating per cell population (Supplemental Fig. 1 and Fig. 1). The percentage of all respective cell populations was determined from live CD45^+^ lung leukocytes after excluding debris and doublets. This percentage was multiplied by the respective total lung cell numbers to determine specific cell population numbers for each animal.

### Lung histopathology and post-translational modifications

Following removal of BALF and blood from the pulmonary vasculature, left lungs were excised and inflated to 15 cm H_2_O pressure with 10% formalin (Fisher Scientific, Fair Lawn, NJ) for 24 h to preserve pulmonary architecture [18]. Fixed left lung lobes were then placed into cassettes, embedded in paraffin, cut (4–5 μm) at midpoint sections to include regions of both large and small airways as well as blood vessels, and stained with hematoxylin and eosin (H&E) or preserved for subsequent IHC. H&E-stained slides of entire lung sections from each animal were reviewed at all scanning magnifications and semi-quantitatively scored for the degree and distribution of lung inflammation. Scores were generated by an expert pathologist blinded to treatment conditions utilizing a previously published scoring system (scored 0 to 4) [18, 33] that evaluates the spectrum of inflammatory changes for alveolar and bronchiolar compartments with higher scores indicating greater inflammation.

Lung sections were also stained with modified Masson’s Trichrome and scanned by Aperio scanner (Leica Biosystems, Deer Park, IL). The VENTANA image viewer (version 3.1.4; Roche Diagnostics, Indianapolis, IN) was utilized to capture 10 images per lung section at 20 × from scanned images. Collagen content in trichrome images was quantified as previously described [12, 34] using Image J FIJI plugin (version: 2.9.0/1.53t U.S. National Institutes of Health, Bethesda, MD).

To quantify CCR2^+^ expression of inflammatory monocyte/macrophages, lung sections were stained with anti-CCR2 (1:100, NBP267700, Lot HM0537, Novus, Littleton, CO) and cross absorbed with donkey anti-rabbit (AlexaFluor488, A21206, Lot #2,156,521, Thermo Fisher, Waltham, MA) at 1:100 and processed as previously described [12]. Slides were mounted with VECTASHIELD® Antifade Mounting Medium with DAPI (4′6-diamindino-2-phenylindole; to identify nuclei)(Cat#H-1200, Lot#ZG1014, Curlingame, CA). Using a Zeiss fluorescent microscope (Zeiss Observer.Z1 Zeiss, White Plains, NY), photographs (10 lung images per mouse) of lung parenchyma were taken, and CCR2^+^ expression by integrated density was quantified by Image J FIJI plugin.

Citrullinated (CIT) and malondialdehyde acetaldehyde (MAA) modified proteins and vimentin were stained [17]. Increased in the context of inflammatory lung diseases, vimentin is an extracellular matrix protein that is also targeted by post-translational modifications generated during the process of inflammation and increased oxidative stress. Prior studies by our group have demonstrated robust co-localization of MAA and CIT with vimentin in lung tissues of mice and humans with inflammatory arthritis and lung disease [17, 35]. Lung sections were stained with Cy5 rabbit anti-vimentin (Bioss, Woburn, MA, USA, 1:100), Zenon AF 594 label (Invitrogen, Carlsbad, CA, USA) and rabbit polyclonal IgG antibody to MAA [17], or a mouse monoclonal anti-peptidyl-citrulline antibody (clone F95 IgMκ, Millipore Sigma, Burlington, MA, USA). Detection of the F95 antibody was done using an AF 488 AffiniPure donkey anti-mouse IgM, µ chain specific antibody (Jackson Immunoresearch, West Grove, PA, USA). DAPI (4′,6-diamidino-2-phenylindole; to identify nuclei) was added and samples were sealed with Fluormount-G (Southern Biotech, Birmingham, AL, USA). Fluorochromes detected using a Revolve fluorescent microscope (ECHO, San Diego, CA, USA). Images were quantified using ImageJ, and colocalization was performed using the Image J (RRID:SCR_003070) FIJI plugin Coloc 2 [17, 18].

### Statistical analysis

Sample-size requirements were extrapolated from previous work assessing post-LPS lung recovery treatments in C57BL/6 [12]. The mean (± SD) of CD11c^int^CD11b^hi^ transitioning/recruited monocyte-macrophages was 0.26 × 10^5^ (0.09 × 10^5^) with saline and 6.5 × 10^5^ (2.2 × 10^5^) with LPS treatment 48 h post-exposure; thus, a sample size of *N* = 2 in each group would achieve 80% power at the 0.05 level of significance to determine an influx of these cells following inflammatory agent exposure as compared to saline control. Experimental groups for the CCR2 trafficking studies include at least 2 mice for each group. The maximum sample sizes for the CCR2^RFP/+^ trafficking studies are *N* = 19 (saline/Sal), *N* = 9 (ODE), *N* = 8 (LPS), and *N* = 5 (PGN). A sample size of *n* = 5 would achieve 80% power at the 0.05 level of significance to detect a 60% reduction in CD11c^int^CD11b^hi^ transitioning/recruited monocyte-macrophages with depletion strategies (i.e., CCR2 KO and clodronate liposomes). For the CCR2 WT vs. KO studies, *N* = 5 (CCR2 WT-Sal), *N* = 5 (CCR2 KO-Sal), *N* = 9 (CCR2 WT-LPS) and *N* = 9 (CCR2 KO-LPS); and for the clodronate (Clod) versus vehicle (Veh) liposome targeted studies, *N* = 8 (Veh + Sal), 8 (Clod + Sal), 8 (Veh + LPS), and 9 (Clod + LPS). Experimental groups for those studies include at least 5 mice for each group. Numbers less than the maximum number reflect limitations in available sample quantity or quality.

Data are presented as the mean ± standard deviation (± SD) with scatter plots depicted for each data point. The Shapiro–Wilk test was utilized to test for normality among treatment groups. If the normality condition was satisfied, parametric statistical tests (one-way ANOVA with subsequent Tukey’s multiple comparison test), and if not satisfied, nonparametric statistical (Kruskal–Wallis with subsequent Dunn’s multiple comparison test) were used to assess differences between any two groups. All statistical analyses were performed using GraphPad Prism (version: 10.1.1) software and statistical significance accepted at a *p* value < 0.05.

### Ethics statement

This study was conducted and reported in accordance with ARRIVE guidelines (https://arriveguidelines.org). All animal procedures were also approved by the University of Nebraska Medical Center (UNMC) Institutional Animal Care and Use Committee and were in accordance with the NIH guidelines for the use of rodents.

## Results

### Inhalant exposures to organic dust extract (ODE), lipopolysaccharide (LPS), and peptidoglycan (PGN) induce lung infiltration of CCR2^+^ monocyte-macrophage (Mɸ) and monocyte subpopulations

In the first set of experiments, heterozygote CCR2^RFP/+^ mice were treated once with ODE (25%), LPS (10 μg), PGN (100 μg), or sterile saline with lung tissue cell infiltrates analyzed at 48 h, as a previous study indicated that this was an optimal time point to detect recruited, infiltrating CD11c^int^CD11b^hi^ transitioning monocytes-macrophages (Mɸ) following acute LPS treatment [12]. There were significant increases (*p* < 0.05) in total cells, CCR2^+^ cells, and CCR2^−^ cells following ODE, LPS, and PGN as compared to saline control with no difference across the treatment groups (Fig. 1A). The 4 monocyte-macrophage subpopulations including alveolar (Alv) Mɸ (CD11c^+^CD11b^lo^), activated (Act) Mɸ (CD11c^+^CD11b^hi^), transitioning monocyte-Mɸ (CD11c^int^CD11b^hi^), and monocytes (CD11c^−^CD11b^hi^) were delineated as previously described [12], with representative contour plots shown in Fig. 1B. CCR2 RFP^+^ and CCR2 RFP^−^ expression in each of the four monocyte/Mɸ subpopulations by treatment condition are depicted in Fig. 1C. CCR2 expression was absent on the Alv Mɸ and Act Mɸ subpopulations but was present on transitioning monocyte-Mɸ and monocyte subpopulations with numbers of these cell subpopulations enumerated in Fig. 1D. The numbers of CCR2^+^ and CCR2^−^ transitioning monocyte-Mɸ were significantly increased with ODE, LPS, and PGN treatment as compared to saline control (*p* < 0.05), but the magnitude of the increase was strikingly greater for the CCR2^+^ (vs. CCR2^−^) transitioning monocyte-Mɸ cells. There were also significant (*p* < 0.05) increases in the numbers of CCR2^+^ and CCR2^−^ monocytes with ODE, LPS, and PGN treatment as compared to saline control with similar magnitude of increases for both CCR2^+^ and CCR2^−^ monocytes. CCR2^−^ Act Mɸ were increased with ODE, LPS, and PGN vs. saline, and correspondingly, CCR2^−^ Alv Mɸ were decreased with ODE, LPS, and PGN vs. saline (Fig. 1D). Although differences vs. saline were demonstrated, there was no difference in the numbers of the monocytes/Mɸ among ODE, LPS, and PGN. Thus, all environmental exposures examined increased CCR2^+^ transitioning monocyte-Mɸ and monocyte subpopulations, but there were also increases in CCR2^−^ monocyte subpopulations and to a lesser degree CCR2^−^ transitioning monocyte-Mɸ.

### Cell surface expression of Ly6C and F4/80 with monocyte/macrophage (Mɸ) subpopulations following inhalant exposures to ODE, LPS, and PGN

**Fig. 2:**
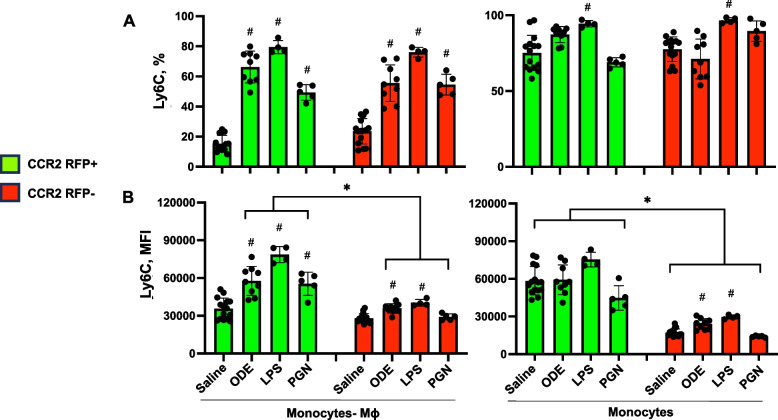
Ly6C expression of monocyte-macrophage (Mɸ) and monocyte subpopulations following organic dust extract (ODE), lipopolysaccharide (LPS), and peptidoglycan (PGN) exposure. C57BL/6 mice were exposed once to ODE (25%), LPS (10 μg), PGN (100 μg), or saline control and euthanized at 48 h. Scatter plots with bars depict mean with SD delineating cells as CCR2 + (green) and CCR2- (red). Expression of Ly6C by percent (**A**) and mean fluorescence intensity (MFI) (**B**) across CD11cintCD11bhi monocyte-Mɸ and CD11c-CD11b + monocyte subpopulations as determined by flow cytometry. Statistical analyses were performed with Kruskal–Wallis with Dunn’s test for multiple comparisons (#*p* < 0.05 vs. respective saline) and (*p < 0.05 denoted by line with brackets denoting difference between same inhalant exposure by CCR2 RFP positive vs. negative). *N* = 15 (saline), 9 (ODE), 4 (LPS), 5 (PGN)

Cell surface expression of Ly6C, a predominant marker of monocytes and/or associated with pro-inflammatory and pro-fibrotic properties [36] by percent expression and mean fluorescence intensity (MFI) were also investigated and summarized (Fig. 2). Ly6C expression was low (< 5%) on Sal-Alv Mɸ and ODE-, LPS-, PGN-Act Mɸ (data not shown). In contrast, Ly6C percent and MFI expression were increased on both CCR2^+^ and CCR2^−^ ODE-, LPS-, PGN- induced CD11c^int^CD11b^hi^ monocyte-Mɸ cells vs. saline control (except MFI expression was not increased for these CCR2^−^ cells following PGN exposure) (Fig. 2 A, B). Moreover, Ly6C MFI expression was significantly (*p* < 0.05) increased on these CCR2^+^ monocyte-Mɸ cells associated with ODE, LPS, and PGN exposure as compared to the corresponding CCR2^−^ monocyte-Mɸ cells. Ly6C percent expression was high on all monocyte populations with a significant (*p* < 0.05) increase with LPS-associated CCR2^+^ and CCR2^−^monocytes vs. saline (Fig. 2A, B). There was an increase in Ly6C MFI expression with ODE and LPS CCR2^−^ monocytes vs. saline with no difference in intensity of the MFI expression across CCR2 RFP^+^ monocytes. As observed with transitioning monocyte-Mɸ cells, MFI expression was increased in all CCR2^+^ monocytes as compared to CCR2^−^ monocytes. These studies demonstrate that Ly6C expression was increased in the recruited CCR2^+^ cells as well as CCR2^−^ cells following exposure to environmental agents, and as such, Ly6C alone may not discriminate monocyte-macrophage subpopulations.

The monocyte and macrophage marker F4/80 (ADGRE1) [37] was also investigated across cell subpopulations (Supplemental Fig. 2). The percent F4/80 expression was ubiquitous across Sal-Alv Mɸ and ODE-, LPS-, PGN-Act Mɸ subpopulations, but the expression intensity by MFI was increased in the ODE-, LPS-, PGN-Act Mɸ vs. Saline-Alv Mɸ (Supplemental Fig. 2). The percent F4/80 expression was high (~ 60–80%) in the transitioning CCR2^+^ and CCR2^−^ CD11c^int^CD11b^hi^ monocyte-macrophage cells (~ 60–80%) and more variable with CD11c^−^CD11b^hi^ monocytes.

###  Inhalant exposures to ODE and LPS induce lung infiltration of CCR2 +NK cells and T cells in addition to CCR2 − neutrophils, B cells, and T cells

**Fig. 3:**
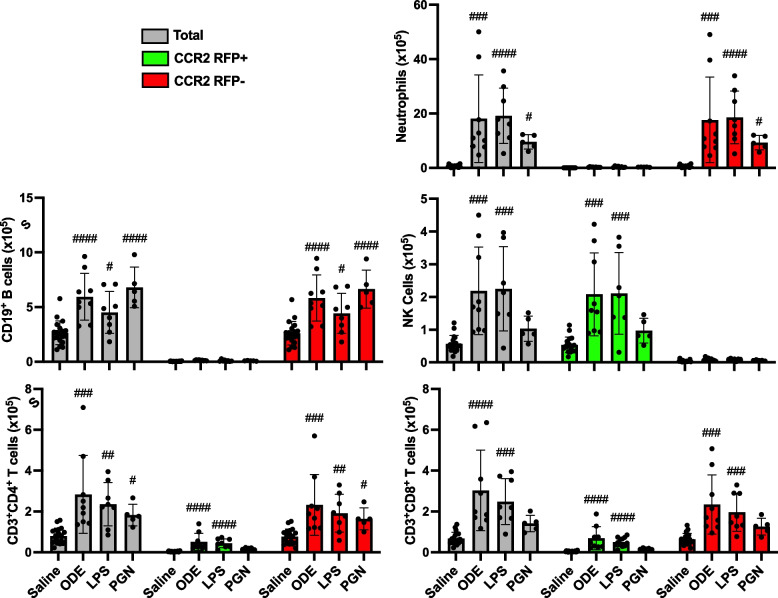
Organic dust extract (ODE) and lipopolysaccharide (LPS) inhalation induce lung infiltration of CCR2 + NK and T cells. C57BL/6 mice were exposed once to ODE (25%), LPS (10 μg), PGN (100 μg), or saline control and euthanized at 48 h. Scatter plots with bars depict mean with SD delineating cells as total (gray), CCR2 + (green), and CCR2- (red). CD11c-Ly6G + neutrophils, CD19 + B cells, CD3-NK1.1 + NK cells, CD3 + CD4 + T cells, and CD3 + CD8 + T cell infiltrates determined by flow cytometry on live CD45 + cells after exclusion of debris and doublets with lung cell % populations multiplied by total lung cells enumerated from lung sample. Gating strategy depicted in Supplemental Fig. 1. Statistical analyses were performed with Kruskal–Wallis with Dunn’s test for multiple comparisons (#*p* < 0.05, ##*p* < 0.01, ###*p* < 0.001, ####*p* < 0.0001) vs. respective saline. *N* = 19 (saline), 9 (ODE), 8 (LPS), 5 (PGN)

The number of CCR2^+^ and CCR2^−^ neutrophils and lymphocytes was also investigated to capture any non-monocyte/macrophage cell-specific CCR2 expression events at 48 h post environmental agent exposure (Fig. 3). CCR2 is recognized to be expressed with NK cells [38] and activated T cells [39]. Indeed, there were significant (*p* < 0.05) increases in CCR2^+^ NK cells, CD3^+^CD4^+^ T cells, and CD3^+^CD8^+^ T cells with ODE and LPS but not PGN treatment vs. saline. There were also significant (*p* < 0.05) increases in CCR2^−^ CD3^+^CD4^+^ T cells and CCR2^−^ CD3^+^CD8^+^ T cells. ODE, LPS, and PGN treatment did not increase CCR2^+^ neutrophils or CD19^+^ B cells. ODE, LPS, and PGN treatment also increased CCR2^−^ neutrophils and CD4^+^ T cells, and ODE and LPS (but not PGN) increased CCR2^−^ CD8^+^ T cells.

### Inhalant LPS-induced lung inflammatory responses are not reduced in CCR2 knock-out (KO) mice

Because there were no major differences in ODE-, LPS-, and PGN-induced CCR2^+^ monocyte-macrophage lung cell infiltrates, LPS was utilized as the prototype environmental inflammatory agent for the remainder of the studies. It was hypothesized that CCR2 knock-out (KO) mice would be protected against LPS-induced lung inflammatory and pro-fibrotic responses due to reduction in the recruitment of transitioning CCR2^+^ monocyte-Mɸ infiltrates. Although there were significant (*p* = 0.0005) reductions (54% reduction) in LPS-induced CD11c^int^CD11b^hi^ monocyte-Mɸ cells, there were no significant reductions in LPS-induced pro-inflammatory and pro-fibrotic mediators in lung homogenates including TNF-α, IL-6, CXCL1, MMP-3, MMP-8, and TIMP-1 (Table 1) and lung histopathology (data not shown) in CCR2 KO mice vs. WT mice. There were also no differences between CCR2 KO and WT mice for LPS-induced total cells, neutrophils, lymphocytes, and macrophages in BALF as well as LPS-induced lung infiltrates including activated Mɸ, monocytes, T and B lymphocytes, and NK cells. In contrast, LPS-induced lung neutrophils were increased in CCR2 KO (vs. WT) mice, and moreover, there were corresponding, likely compensatory, increases in lung and serum CCL2 and CCL7, chemoattractants that predominately drive monocyte recruitment but also can affect lymphocytes and neutrophils, with the LPS treated CCR2 KO mice.

**Table 1:** Inhalant LPS-induced lung inflammatory responses are not reduced in CCR2 knock-out (KO) mice

	**WT-Saline**	**KO-Saline**	**WT-LPS**	**KO-LPS**
**BALF cells (× 10** ^**5**^ **)**				
Total Cells	0.85 ± 0.4	0.96 ± 0.24	16.45 ± 6.39^#^	28.47 ± 19.77^#^
Neutrophils	0.42 ± 0.19	0.42 ± 0.23	9.71 ± 3.38^#^	17.35 ± 12.46^#^
Mɸ	0.78 ± 0.39	0.84 ± 0.19	3.11 ± 1.62	3.45 ± 1.56
Lymphocytes	0.04 ± 0.07	0.02 ± 0.04	0.09 ± 0.14	0.08 ± 0.12
**Lung cells (× 10** ^**5**^)				
Total cells	8.69 ± 3.01	7.86 ± 1.99	33.08 ± 7.41^#^	43.05 ± 15.18^#^
Neutrophils	0.42 ± 0.19	0.48 ± 0.25	8.48 ± 2.47^#^	**18.94 ± 9.22** ^#,*^
Alveolar Mɸ	2.01 ± 0.88	3.26 ± 1.61	0.35 ± 0.25^#^	0.30 ± 0.24^#^
Activated Mɸ	0.15 ± 0.09	0.22 ± 0.08	2.27 ± 0.34^#^	1.91 ± 0.45^#^
Monocyte-Mɸ	0.34 ± 0.15	0.27 ± 0.15	4.77 ± 1.9^#^	**2.20 ± 0.82** ^#,*^
Monocytes	0.28 ± 0.06	0.20 ± 0.10	1.51 ± 0.57^#^	1.13 ± 0.77^#^
CD19^+^ B cells	2.14 ± 0.74	1.81 ± 0.53	4.81 ± 1.08^#^	5.01 ± 0.78^#^
CD4^+^ T cells	0.98 ± 0.38	0.60 ± 0.18	2.44 ± 0.85^#^	2.66 ± 0.82^#^
CD8^+^ T cells	0.94 ± 0.41	0.69 ± 0.23	3.08 ± 0.99^#^	3.43 ± 0.91^#^
NK Cells	0.94 ± 0.41	0.66 ± 0.25	2.95 ± 1.04^#^	2.52 ± 1.00^#^
**Lung Mediators**				
TNF-⍺ (pg/ml)	1.54 ± 2.22	0.00 ± 0.00	26.52 ± 13.66^#^	33.54 ± 27.50^#^
IL-6 (pg/ml)	3.00 ± 1.88	3.90 ± 2.34	67.26 ± 37.0^#^	77.41 ± 56.05^#^
CXCL1 (pg/ml)	37.60 ± 27.99	55.3 ± 58.27	642.8 ± 274.7^#^	799.8 ± 186^#^
MMP-3 (ng/ml)	5.08 ± 1.69	4.70 ± 1.00	61.85 ± 35.15^#^	77.91 ± 33.05^#^
MMP-8 (ng/ml)	26.74 ± 7.45	21.97 ± 17.59	102.69 ± 36.01^#^	117.49 ± 57.75^#^
TIMP-1 (ng/ml)	1.15 ± 0.73	2.39 ± 2.58	30.16 ± 10.21^#^	27.70 ± 15.36^#^
CCL2 (pg/ml)	31.59 ± 7.62	134.7 ± 74.02	707 ± 65^#^	**1730 ± 840** ^#,*^
CCL7 (pg/ml)	21.21 ± 9.16	62.87 ± 32.28	1652 ± 420.7^#^	3655 ± 1662^#^
**Serum**				
CCL2 (pg/ml)	36.4 ± 23.0	141.4 ± 35.9	52.09 ± 18.2	**250 ± 108** ^#*^
CCL7 (pg/ml)	101.4 ± 27.4	**3420 ± 908.5** ^**#**^	141.1 ± 56.4	**5530 ± 1129** ^#*^

Mean ± SD

^#^ *p* < 0.05 vs. saline,

^*^ *p* < 0.05 CCR2 WT vs. KO & **bold**

Mice/group: *N* = 5 (Saline) and *N* = 9 (LPS)

###  LPS-induced lung transitioning, infiltrating CD11c int CD11b hi are reduced with systemic delivery of clodronate liposomes

**Fig. 4:**
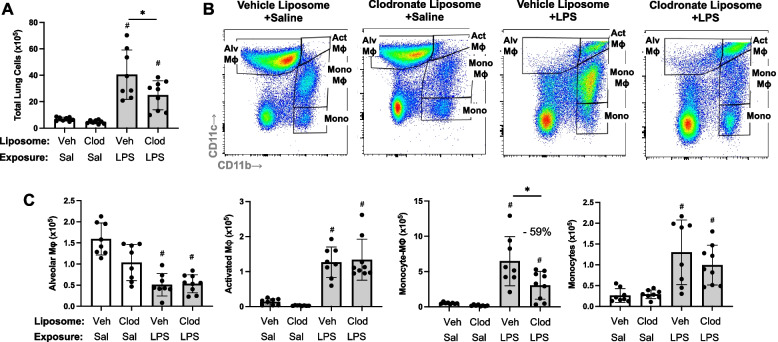
LPS-induced transitioning CD11c^int^CD11b^hi^ monocytes/macrophages are reduced with systemic delivery of clodronate liposomes. Mice were pre-treated with vehicle (Veh) or clodronate (Clod) liposomes 24 h prior to a one-time treatment with LPS (10 μg) or saline (Sal) control and euthanized at 48 h. Scatter plot with bars depicting mean with SD. **A** Total lung cells enumerated. **B** Representative contour plot of the four monocyte (mono)-Mɸ subpopulations across groups based upon CD11c and CD11b expression after removal of neutrophils gated from live CD45^+^ cells after excluding debris and doublets. **C** Number of CD11c^+^CD11b^lo^ alveolar (Alv) Mɸ, CD11c^+^CD11b^hi^ activated (Act) Mɸ, CD11c^int^CD11b^hi^ mono-Mɸ, and CD11c^−^CD11b.^hi^ monocytes determined by multiplying lung cell % population by total lung cells enumerated from lung sample. (#*p* < 0.05 vs. respective saline) and (*p < 0.05 denoted by line with brackets denoting difference between groups). *N* = 8 (Veh + Sal), 8 (Clod + Sal), 8 (Veh + LPS), 9 (Clod + LPS)

In an alternative approach to deplete recruited lung monocytes-macrophages induced by environmental exposures, intravenous clodronate liposomes (vs. vehicle control liposomes) were dosed one day prior to LPS (and saline) treatment to reduce circulating/systemic reservoir of available monocytes-macrophages with mice euthanized at 48 h following LPS exposure. In these studies, there was a reduction in total lung cells in tissue homogenates associated with a 59% reduction in LPS-induced lung CD11c^int^CD11b^hi^ monocyte-Mɸ infiltrates in clodronate liposome pre-treated mice compared to control-treated with mice (Fig. 4). In contrast, there were no treatment differences in the number of LPS-induced Alv Mɸ, Act Mɸ or monocytes. There were also no treatment differences in the number of inflammatory cells in BALF and no treatment differences in the number of, CD19^+^ B cells, CD4^+^ and CD8^+^ T cells, and NK cells in tissue homogenates following LPS exposure (Supplemental Table 1).

###  Effects of systemic delivery of clodronate liposomes with LPS-induced lung inflammation, collagen deposition, and infiltrating CCR2 + cells

**Fig. 5:**
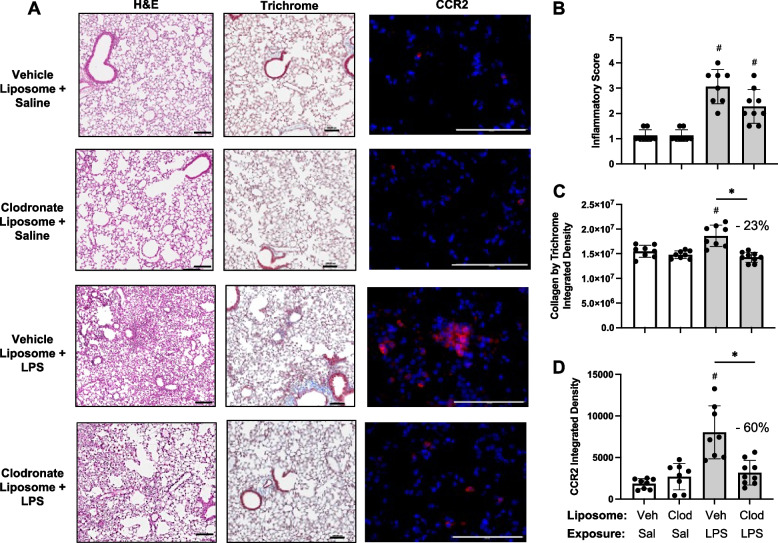
Effects of systemic clodronate liposome delivery with LPS-induced lung inflammation, collagen, and infiltrating CCR2^+^ cells. Mice were pre-treated with vehicle (Veh) or clodronate (Clod) liposomes 24 h prior to a one-time treatment with LPS (10 μg) or saline (Sal) control and euthanized at 48 h. **A** Representative images from treatment groups stained by H&E, trichome, and CCR2 (red) with DAPI nuclei staining (blue) by confocal microscopy. Scatter plots with bars depict mean with SD of semi-quantitative lung inflammatory score (**B**) and integrated density of collagen (**C**), and CCR2 (**D**) quantified per each mouse. Statistical analyses were performed with Kruskal–Wallis with Dunn’s multiple comparison (inflammatory scores) and ANOVA with Tukey’s multiple comparison (collagen content and CCR2) (#*p* < 0.05 vs. respective saline) and (*p < 0.05 denoted by line with brackets denoting difference between groups). *N* = 8 (Veh + Sal), 8 (Clod + Sal), 8 (Veh + LPS), 9 (Clod + LPS). Line scale denotes 100 μm

Lung sections from these same mice pre-treated with vehicle and clodronate liposomes followed by saline and LPS challenge were evaluated for histopathological changes by H&E, collagen deposition by trichrome staining, and CCR2^+^ cell infiltrates (Fig. 5A). Although semi-quantitative inflammatory scores following LPS exposure were reduced with clodronate liposome pre-treatment compared to vehicle control (Fig. 5B), this difference did not reach statistical significance. LPS-induced collagen deposition was reduced by 23% (*p* < 0.05) with clodronate liposome pretreatment (Fig. 5C). Moreover, LPS-induced CCR2^+^ cell infiltrates were reduced by 60% (*p* < 0.05) with clodronate liposome pretreatment (Fig. 5D), consistent with reductions observed in CD11c^int^CD11b^+^ monocyte- Mɸ demonstrated by flow cytometry.

### LPS-induced lung pro-fibrotic and inflammatory mediators modulated following systemic delivery of clodronate liposomes

**Fig. 6:**
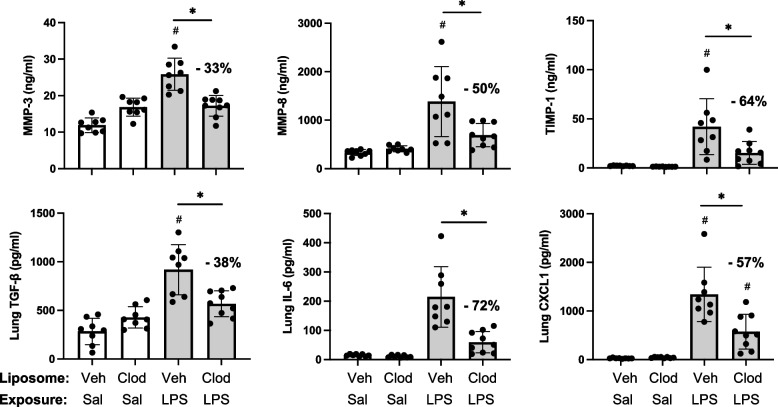
LPS-induced lung pro-fibrotic and inflammatory mediators modulated following systemic delivery of clodronate liposomes. Mice were pre-treated with vehicle (Veh) or clodronate (Clod) liposomes 24 h prior to a one-time treatment with LPS (10 μg) or saline (Sal) control and euthanized at 48 h. Scatter plots with bars depict mean with SD of protein levels of matrix metalloproteinase (MMP)-3, MMP-8, metalloproteinase inhibitor (TIMP-1), transforming growth factor (TGF)-β, IL-6, and neutrophil chemokine CXCL1 of lung homogenate. Statistical analyses were performed with ANOVA and Tukey’s multiple comparison test with significance (#*p* < 0.05 vs. respective saline) and (*p < 0.05 denoted by line with brackets denoting difference between groups) with % reduction noted. *N* = 8 (Veh + Sal), 8 (Clod + Sal), 8 (Veh + LPS), 9 (Clod + LPS)

Pre-treatment with intravenous clodronate liposomes (vs. vehicle) also resulted in significant reductions in LPS-induced levels of pro-fibrotic mediators in lung homogenates including MMP-3 (33% reduction), MMP-8 (50% reduction), TIMP-1 (64% reduction), and TGF-β (38% reduction) (Fig. 6). Moreover, there were also significant (*p* < 0.05) reductions in LPS-induced pro-inflammatory mediators including IL-6 (72% reduction) and neutrophil chemoattractant CXCL1 (57% reduction) with clodronate (vs. vehicle) liposome pre-treatment (Fig. 6). Lung levels of TNF-α induced by LPS exposure were not reduced with clodronate liposome pre-treatment, and there were also no differences in LPS-induced lung CCL2 and CCL7 levels between clodronate and vehicle liposome pre-treatment (Supplemental Table 1).

### LPS-induced lung CIT and MAA autoantigens and vimentin expression were reduced with systemic delivery of clodronate liposomes

**Fig. 7:**
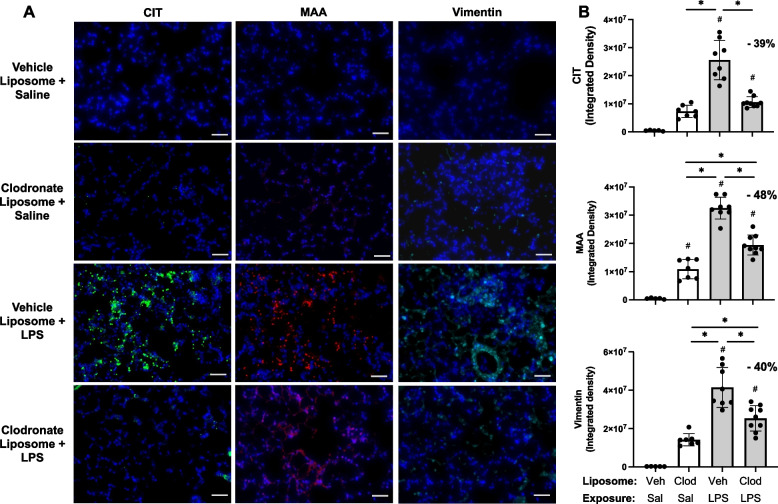
LPS-induced lung CIT and MAA autoantigens and vimentin expression decrease with systemic clodronate liposome delivery. Mice were pre-treated with vehicle (Veh) or clodronate (Clod) liposomes 24 h prior to a one-time treatment with LPS (10 μg) or saline (Sal) control and euthanized at 48 h. **A** Representative confocal microscopy images of lung tissue from treatment groups stained for citrulline (CIT, green) and malondialdehyde-acetaldehyde (MAA, red) modified proteins and vimentin (teal). Line scale denotes 70 μm. **B** Scatter plots with bars depict mean with SD of integrated density of CIT- and MAA-modified proteins and vimentin quantified per each mouse. Statistical analyses were performed with ANOVA with Tukey’s for multiple comparisons (#*p* < 0.05 vs. respective saline) and (*p < 0.05 denoted by line with brackets denoting difference between groups). *N* = 5 (Veh + Sal), 7 (Clod + Sal), 8 (Veh + LPS), 9 (Clod + LPS)

Based upon findings of decreased pro-fibrotic mediators and prior findings demonstrating that repetitive inhalant environmental exposures induce post-translational changes implicated in inflammatory and fibrotic lung disease [17, 18], lung tissues were stained for CIT- and MAA-modified antigens as well as vimentin. CIT- and MAA-modified proteins and vimentin were significantly increased following a one-time LPS exposure vs. saline control at 48 h post-LPS exposure (Fig. 7A, B). Moreover, there were significant (*p* < 0.05) reductions in LPS-induced lung CIT-modified protein expression (39% reduction), MAA-modified protein expression (48% reduction), and vimentin (40% reduction) with clodronate (vs. vehicle) liposome administration.

## Discussion

Lung disease represents a major cause of occupation-related illness for which therapeutic approaches to alleviate disease burden are lacking. Recruited, infiltrating, and transitioning monocytes-macrophages have been implicated as critical cells in the immunopathogenesis of chronic lung disease. Here, our preclinical animal studies first defined and compared the trafficking of the inflammatory CCR2^+^ monocytes/macrophages to the lung following organic dust, endotoxin, and peptidoglycan exposure. These studies demonstrated striking increases in CCR2^+^ recruited/transitioning CD11c^int^CD11b^hi^ monocyte-Mɸ and CD11c^−^CD11b^hi^ monocyte subpopulations as well as striking increases in CCR2^−^ monocytes with these environmental exposures. However, CCR2 KO mice were not protected against inflammatory responses induced following endotoxin exposure despite a reduction (54%) in exposure induced CCR2^+^ recruited/transitioning CD11c^int^CD11b^hi^ cells. Instead, systemic depletion of monocytes by intravenous clodronate liposome administration was associated with not only a reduction (59% reduction) in these transitioning monocyte-macrophages, but also a corresponding reduction in endotoxin-induced collagen deposition, extracellular matrix release, vimentin, and lung autoantigen expression. Thus, preventing the influx of circulating/recruited monocytes to the lung, as opposed to specifically targeting CCR2, following environmental exposures may represent a strategic area to further develop to reduce disease burden in occupationally exposed at-risk persons.

Occupational and environmental exposures are inherently complex, with highly concentrated exposures commonplace in a variety of settings, which can initiate and perpetuate the development of lung disease. In our studies aimed at defining the trafficking of monocytes-macrophages to the lung, we observed similar effects following separate exposures to high concentrations of complex organic dust extract (ODE), LPS, and PGN, all of which resulted in a robust increase in monocyte-macrophage recruitment. This supports the current focus of therapeutic approaches for environmental and occupational-associated diseases that have centered on specific agent identification and risk reduction measures and mitigation [40]. Supporting the relevance of experiments focused on high exposure doses, endotoxin levels encountered in real-world settings are highly variable and often exceed occupational exposure limits in agriculture settings by several orders of magnitude [41, 42].

Lung monocytes-macrophages are important in mediating the response to inflammatory bioaerosol exposure, and recruited/transitional monocytes-macrophages specifically are implicated in the transition of acute inflammation to lung fibrosis in animal models and clinical investigations [43]. CCR2-expressing leukocytes are required for the progression of bleomycin-induced fibrosis, as CCR2 KO mice were protected against collagen deposition, macrophage infiltration, and MMP deposition in this model [44, 45]. Likewise, pre-treatment with a CCR2 antagonist reduced lung fibrosis in a mouse model of scleroderma [46]. Although lung injury promotes the release of CCL2 by several different lung cell types, including airway epithelial cells, and serves as a chemoattractant for CCR2 pro-fibrotic macrophages [47, 48], CCL2 neutralizing antibodies did not alter disease progression or mitigate lung function decline in patients with idiopathic pulmonary fibrosis, but instead was associated with an increase in endogenous CCL2 expression and other adverse compensatory changes [49]. We also found that CCL2 levels were increased in LPS-exposed CCR2 KO mice. The lack of efficacy observed with CCL2 targeting is consistent with our results in which CCL2 KO mice did not appear to be meaningfully protected from LPS-induced lung injury.

In endotoxin exposure models of lung injury, several studies have also demonstrated that there is a decrease in the endotoxin-induced recruitment of peripheral blood monocytes, exudative macrophages, F4/80^+^ lung cells, lung neutrophils, and lung cell infiltrates in CCR2 KO mice as well as mice depleted of systemic monocytes by clodronate liposomes [25, 50–54]. Our studies confirm the reduction in infiltrating monocytes/macrophages with both the CCR2 KO and the administration clodronate liposomes, but also simultaneously highlight striking differences in these modeling approaches to reduce profibrotic and inflammatory responses following LPS exposure. In addition to finding no meaningful benefit for globally depleting CCR2-expressing cells in endotoxin-induced lung disease, we demonstrated that CCR2 KO mice had increased neutrophil accumulation with compensatory increases in CCL2 and CCL7 following LPS exposure, suggesting this strategy may actually yield detrimental effects.

Our observations are consistent with a study by Gurczynski and colleagues demonstrating no protection against ɣ-herpesvirus-induced pneumonitis and fibrosis with CCR2 KO mice and that CCR2^+^ cells played a suppressive role by limiting collagen and IL-17 production [55]. Another potential compensatory mechanism supported by this study is that the environmental exposures induced the expression of CCR2^+^ on the CD4^+^ and CD8^+^ T-cell infiltrates. Others have demonstrated suppressive roles for CCR2-expressing T cells in lung infections and inflammatory responses [56]. Moreover, CCR2 is highly expressed on NK cells, and both ODE and LPS induced the recruitment of NK cells. Thus, depletion of CCR2 function with lymphocytes may have negated any potential benefit of reducing CCR2^+^ monocytes-macrophages. Based on these findings, further investigations are warranted to understand the functional role of CCR2 on infiltrating lymphocytes in the setting of environmental exposure-induced lung disease.

In contrast to CCR2 targeting, the strategy of inhibiting the recruitment of peripheral blood monocytes to the lung through the systemic administration of clodronate liposomes demonstrated benefit with most, but not all, endpoints examined. Beneficial responses are best characterized by a reduction in the pro-fibrotic properties of lung monocytes-macrophages. Namely, endotoxin-induced collagen deposition, MMP3, MMP8, TIMP-1, TGF-β, IL-6, and CXCL1 (but not TNF-⍺) were reduced by depleting peripheral blood monocytes. This corresponded with reductions observed in CIT- and MAA-modified protein generation following endotoxin exposure. This is relevant, as proteins co-modified with CIT and MAA stimulate undifferentiated macrophages towards a mixed M1/M2 phenotype [57] and secrete soluble factors that drive an aggressive fibroblast phenotype [20]. Whereas there was no compensatory increase in CCL2 or CCL7 release following the administration of clodronate liposomes as observed with CCR2 KO mice, neither strategy appeared to mitigate the histopathologic changes initiated by acute LPS exposure.

These collective findings underscore the importance of the recruited peripheral blood monocyte transitioning to a lung monocyte/macrophage population in mediating pro-fibrotic processes in the lung as well as generation of post-translationally modified proteins following endotoxin exposure. Therefore, depletion and/or inhibition of “recruitable” peripheral blood monocytes may represent a novel strategy to reduce the burden of lung diseases resulting from select environmental exposures. Whereas CCR2 abolition did not exhibit protection against LPS-induced inflammatory or pro-fibrotic responses, antagonism of other monocyte trafficking receptors (i.e., CCR1, 5, 6, 7), blockade of adhesion molecules (i.e., selectins, integrins, ICAM-1/VCAM-1), or inhibition of chemokines (i.e., CCL2, CCL5, CCL7) may demonstrate therapeutic benefit given depletion of the recruitable monocyte reservoir demonstrated protective effects in our model of acute exposure-induced lung inflammation [58–60]. Moreover, understanding the mechanisms governing the crosstalk between lung monocytes and airway structural cells including fibroblasts/myofibroblasts is warranted.

In conclusion, high concentration exposure to environmental and occupational exposures including agricultural organic dust extracts, endotoxin, and peptidoglycan induce the recruitment of CCR2^+^ and CCR2^−^ peripheral blood monocytes transitioning to lung resident monocytes/macrophages. Depleting peripheral blood monocytes by systemic administration of clodronate liposomes, but not through CCR2 KO animal strategies, resulted in the reduction of endotoxin-induced pro-inflammatory and pro-fibrotic mediators. Developing translational strategies to reduce the recruitment of these cells following exposures may be warranted to reduce risk of lung disease.

### Supplementary Information

**Additional file 1: Supplemental Fig. 1.** Gating strategy for identification of non-debris, singlets, live CD45^+^ myeloid and lymphoid cells. For flow analysis, all panels were first gated as forward scatter-area (FSC-A) x side scatter-area (SSC-A) to omit debris, dead, or apoptotic cells. This was followed by two single cell gates to omit doublets (FSC-A x FSC-heigh (H) and SSC-A x SSC-H), followed by live/dead gate and then CD45 gate to assure removal of any additional dead or apoptotic cells and non-leukocytes. The CD45^+^ cells were placed on a CD11c x Ly6G gate to select Ly6G^+^ neutrophils. Non-neutrophils were gated for CD19^+^ B cells (CD19 x SSC gate). This was followed by non-B cells gated on CD11c x CD11b gate to select CD11c^+^CD11b^lo^ alveolar (Alv) macrophages (Mɸ), CD11c^+^CD11b^hi^ activated (act) Mɸ, CD11c^int^CD11b^hi^ transitioning monocytes (Mono)—Mɸ, and CD11c^−^CD11b^hi^ monocytes (Mono). The negative or non-monocyte/macrophage populations were placed on CD3 x NK1.1 to select CD3^+^ T cells and CD3^−^NK1.1^+^ NK cells, and then a CD4 x CD8 gate to select CD3^+^CD4^+^ and CD3^+^CD8^+^ T cells. A CCR2 RFP x SSC gate is shown for neutrophils and lymphocytes to demonstrate CCR2^+^ staining on specific lung cell subpopulations. Lung sample shown is from an LPS-exposed mouse.

**Additional file 2: Supplemental Fig. 2.** F4/80 (ADGRE1) expression on monocyte-macrophage (Mɸ) subpopulations following organic dust extract (ODE), lipopolysaccharide (LPS), and peptidoglycan (PGN) inhalation exposure. C57BL/6 mice were exposed once to ODE (25%), LPS (10 μg), PGN (100 μg), or saline control and euthanized at 48 h. Scatter plots with bars depict mean with SD delineating cells as CCR2^+^ (green) and CCR2^−^ (red). Expression of F4/80 by percent (A) and mean fluorescence intensity (MFI) (B) across alveolar (Alv) Mɸ, activated (Act) Mɸ, monocyte-Mɸ, and monocyte subpopulations as determined by flow cytometry. Statistical analyses were performed with Kruskal–Wallis with Dunn’s test for multiple comparisons (#*p* < 0.05 vs. respective saline) and (*p < 0.05 denoted by line with brackets denoting difference between same inhalant exposure by CCR2 RFP positive vs. negative). *N* = 15 (saline), 9 (ODE), 4 (LPS), 5 (PGN).

**Additional file 3: Supplemental Table 1.** LPS-induced airway inflammatory indices not affected with systemic delivery of clodronate liposomes.

## Data Availability

Data that support the findings of this study have been deposited in Zenodo. The data are embargoed until manuscript acceptance for publication. The link to the data is below. https://zenodo.org/records/10641513?token=eyJhbGciOiJIUzUxMiJ9.eyJpZCI6IjNjYmNhNDA0LThmMDYtNDBmZS1iMTc2LTZkYTQ1NDZjZjExOCIsImRhdGEiOnt9LCJyYW5kb20iOiIzODkyNWQ0MmUxYzdlNzE4Zjg5YWRkZDVhMzllMTYzNiJ9.KUYVheTKH7IITATY9EiCchsVBH4Db6OoaIi-c2fAakjMpTypBhSTuAHeXMnz-9qoeyVrUzXboA0T7EpsPGMwVw.
