# Supplementary figures and images for: Targeting transitioning lung monocytes/macrophages as treatment strategies in lung disease related to environmental exposures
Source: Respir Res. 2024 Apr 9;25:157. doi: 10.1186/s12931-024-02804-3 (PMC11003126; doi:10.1186/s12931-024-02804-3)

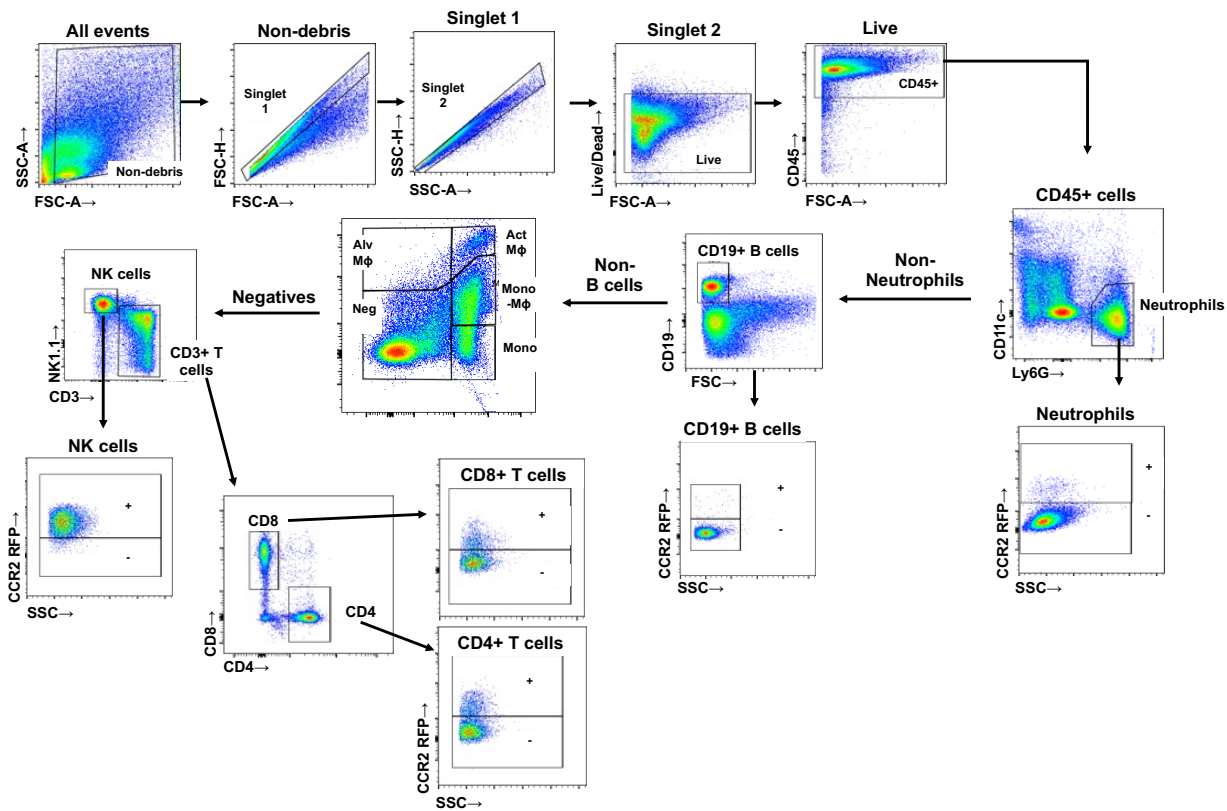

Supplement: Supplementary file 1 — Additional file 1: Supplemental Fig. 1. Gating strategy for identification of non-debris, singlets, live CD45+ myeloid and lymphoid cells. For flow analysis, all panels were first gated as forward scatter-area (FSC-A) x side scatter-area (SSC-A) to omit debris, dead, or apoptotic cells. This was followed by two single cell gates to omit doublets (FSC-A x FSC-heigh (H) and SSC-A x SSC-H), followed by live/dead gate and then CD45 gate to assure removal of any additional dead or apoptotic cells and non-leukocytes. The CD45+ cells were placed on a CD11c x Ly6G gate to select Ly6G+ neutrophils. Non-neutrophils were gated for CD19+ B cells (CD19 x SSC gate). This was followed by non-B cells gated on CD11c x CD11b gate to select CD11c+CD11blo alveolar (Alv) macrophages (Mɸ), CD11c+CD11bhi activated (act) Mɸ, CD11cintCD11bhi transitioning monocytes (Mono)—Mɸ, and CD11c−CD11bhi monocytes (Mono). The negative or non-monocyte/macrophage populations were placed on CD3 x NK1.1 to select CD3+ T cells and CD3−NK1.1+ NK cells, and then a CD4 x CD8 gate to select CD3+CD4+ and CD3+CD8+ T cells. A CCR2 RFP x SSC gate is shown for neutrophils and lymphocytes to demonstrate CCR2+ staining on specific lung cell subpopulations. Lung sample shown is from an LPS-exposed mouse. [file 12931_2024_2804_MOESM1_ESM.pdf]

CCR2 RFP+  
CCR2 RFP-

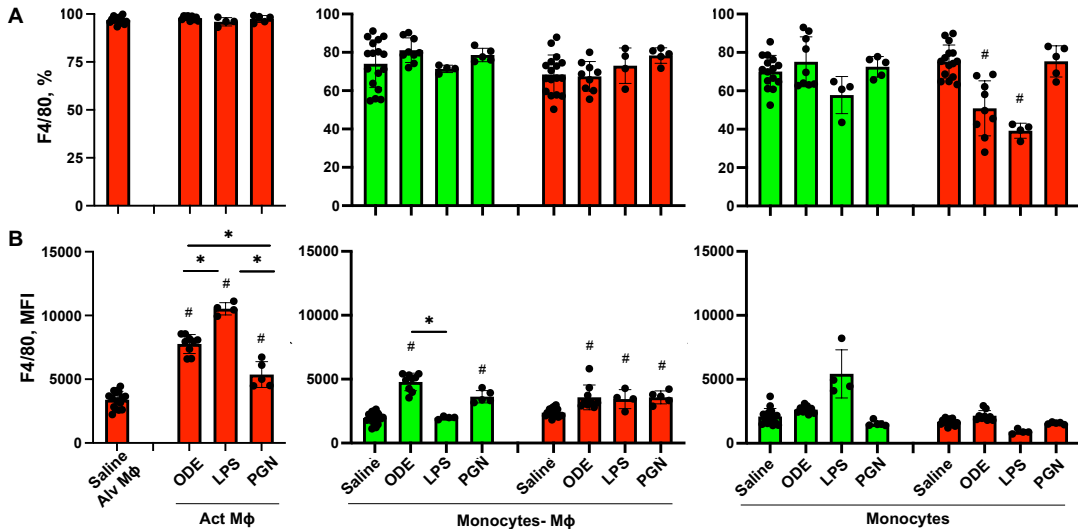

Supplement: Supplementary file 2 — Additional file 2: Supplemental Fig. 2. F4/80 (ADGRE1) expression on monocyte-macrophage (Mɸ) subpopulations following organic dust extract (ODE), lipopolysaccharide (LPS), and peptidoglycan (PGN) inhalation exposure. C57BL/6 mice were exposed once to ODE (25%), LPS (10 μg), PGN (100 μg), or saline control and euthanized at 48 h. Scatter plots with bars depict mean with SD delineating cells as CCR2+ (green) and CCR2− (red). Expression of F4/80 by percent (A) and mean fluorescence intensity (MFI) (B) across alveolar (Alv) Mɸ, activated (Act) Mɸ, monocyte-Mɸ, and monocyte subpopulations as determined by flow cytometry. Statistical analyses were performed with Kruskal–Wallis with Dunn’s test for multiple comparisons (#p < 0.05 vs. respective saline) and (*p < 0.05 denoted by line with brackets denoting difference between same inhalant exposure by CCR2 RFP positive vs. negative). N = 15 (saline), 9 (ODE), 4 (LPS), 5 (PGN). [file 12931_2024_2804_MOESM2_ESM.pdf]
