# Supplementary material for: Targeting transitioning lung monocytes/macrophages as treatment strategies in lung disease related to environmental exposures
Source: Respir Res. 2024 Apr 9;25:157. doi: 10.1186/s12931-024-02804-3 (PMC11003126; doi:10.1186/s12931-024-02804-3)
Supplement: Supplementary file 3 — Additional file 3: Supplemental Table 1. LPS-induced airway inflammatory indices not affected with systemic delivery of clodronate liposomes. [file 12931_2024_2804_MOESM3_ESM.docx]

| **Supplemental Table 1. LPS-induced airway inflammatory indices not affected with systemic delivery of clodronate liposomes.** | | | | |
| --- | --- | --- | --- | --- |
|  | **Veh liposome**  **+ Saline** | **Clod liposome**  **+ Saline** | **Veh liposome**  **+ LPS** | **Clod liposome + LPS** |
| **BALF cells (x10^5^)** |  |  |  |  |
| Total Cells | 1.18±0.87 | 0.70±0.15 | 17.06±3.72^#^ | 15.69±5.07^#^ |
| Neutrophils | 0.13±0.24 | 0.06±0.09 | 14.43±3.17^#^ | 13.06±5.11^#^ |
| Mɸ | 1.00±0.57 | 0.71±0.28 | 1.70±1.27 | 2.13±0.96 |
| Lymphocytes | 0.01±0.01 | 0.002±0.004 | 0.002±0.005 | 0.13±0.20 |
| **Lung cells (x10^5^**) |  |  |  |  |
| Neutrophils | 0.86±0.39 | 0.45±0.14 | 18.65±10.74^#^ | 10.26±5.84^#^ |
| CD19^+^ B cells | 1.39±0.46 | 1.16±0.40 | 3.19±0.89^#^ | 2.20±0.62^#^ |
| CD4^+^ T cells | 0.49±0.15 | 0.389±0.11 | 1.44±0.60^#^ | 1.21±0.63^#^ |
| CD8^+^ T cells | 0.38±0.12 | 0.35±0.10 | 2.28±0.95^#^ | 1.71±0.90^#^ |
| NK Cells | 0.64±0.41 | 0.28±0.08 | 1.54±0.60^#^ | 1.04±0.47 |
| **Lung Mediators** |  |  |  |  |
| TNF-⍺ (pg/ml) | 0.00±0.00 | 0.00±0.00 | 60.75±34.03^#^ | 48.88±28.12^#^ |
| CCL2 (pg/ml) | 30.73±11.0 | 98.44±64.44 | 1369±1331^#^ | 1309±773^#^ |
| CCL7 (pg/ml) | 28.80±10.02 | 25.66±14.06 | 2233±1177^#^ | 1397±1127^#^ |

Mean±SD. #p<0.05 vs. saline

N=8 (Veh+Sal), 8 (Clod+Sal), 8 (Veh+LPS), 9 (Clod+LPS)
